# Editorial: Polymeric microarchitectures for tissue regeneration and drug screening

**DOI:** 10.3389/fbioe.2023.1144991

**Published:** 2023-02-02

**Authors:** Ranjith Kumar Kankala, Yu Shrike Zhang, Lifeng Kang, Luigi Ambrosio

**Affiliations:** ^1^ College of Chemical Engineering, Huaqiao University, Xiamen, Fujian, China; ^2^ Harvard Medical School, Boston, MA, United States; ^3^ Faculty of Medicine and Health, The University of Sydney, Darlington, NSW, Australia; ^4^ National Research Council (CNR), Roma, Lazio, Italy

**Keywords:** microfluidics, biofabrication, tissue engineering, minimally invasive, cell delivery, drug development

Tissue engineering aims to restore malfunctioned tissues by fabricating three-dimensional (3D) biomimetic tissue substitutes that emulate their native counterparts. This field has garnered enormous interest from researchers due to increased organ replacement therapies and the shortage of donors (Langer and Vacanti, 1993). Several advancements have resulted in the generation of highly organized 3D scaffolds to improve the control over the microenvironment for tissue growth, such as biocompatible fibrous scaffolds, photo-cross-linkable hydrogels, and 3D biodegradable porous scaffolds (Martin et al., 2004; Asakawa et al., 2010). Numerous efforts in fabricating artificial tissue constructs have been dedicated to repairing tissue damage and highlighting the significance of vascularization and innervation on tissue maturation (Leijten et al., 2016). Over the past few decades, diverse preclinical screening methods have been explored to demonstrate the pharmacological and toxicological characteristics of various therapeutic drugs. The traditional cell monolayer-based 2D approach often suffers from limitations in recapitulating the highly complex, natural extracellular matrix (ECM)-like microenvironment. Although the 3D scaffold-free cell aggregates emerged as an alternative to 2D monolayers, they fail to recapitulate critical attributes of the natural tumor microenvironment and poor survival rates of cells.

The implantation of bulk scaffolds for tissue repair often utilizes complex surgical procedures, which may generate severe inflammatory reactions resulting in the harsh microenvironment, where the survival of cells remains low (Ferrari et al., 1998; Kankala et al., 2018; Kankala et al., 2019). In this context, several biodegradable polymeric microspheres with highly open and interconnected pores have been reported, as these carriers enable exceptional cell encapsulation efficacy in their entire volume and facilitate their minimally invasive delivery. Notably, these cell-laden microspheres could further aggregate and form microtissues for tissue engineering applications. Utilizing the 3D polymeric microarchitectures harbored with cells offer enormous advantages in terms of effective cell-harboring and carrying capacities, enabling the supply of oxygen and nutrients for cell proliferation (Van Wezel, 1967; Khademhosseini et al., 2006; Jiang et al., 2016). These injectable modularized units of cell-loaded microspheres, cell lamellae, and cell-laden microgels, obtained using various biofabrication strategies, offer easy packing, minimally invasive, and improved cell retention capacity than the direct injection of cells alone (Khademhosseini et al., 2006). Compared to traditional 2D monolayer and 3D cellular spheroids for drug screening, these cellularized polymeric microarchitectures reflect a more accurate tumor microenvironment in cellular interactions and ECM remolding toward drug evaluation and cancer research. In recent times, these innovative polymeric architectures have been explicitly applied to investigate the pharmacological and toxicological characteristics of drugs (Brancato et al., 2017; Pradhan et al., 2017).

This proposed thematic Research Topic entitled “Polymeric Microarchitectures for Tissue Regeneration and Drug Screening” is intended to explore the advancements in various innovative synthetic strategies and plausible mechanistic elucidations towards the development of polymeric microarchitectures for tissue engineering and drug screening applications. In this context, this thematic Research Topic collects articles exploring the opportunities and challenges relevant to scale-up and the clinical translation of these innovative scaffolding systems with considerations of biosafety and degradability. Moreover, this Research Topic is intended to present mechanistic views and understanding of interactions within the biological interfaces to accelerate the efficacy of polymeric microsystems through precise cell delivery and screening of therapeutics at different levels.

Prof. Niu and colleagues demonstrate the fabrication of 3D microscaffolds using the silk fibroin (SF) and chitosan (CS), as well as 1-ethyl-3-(3-dimethylaminopropyl)-carbodiimide (EDC) and sodium tripolyphosphate (TPP) as cross-linking agents (Niu et al., 2022). These EDC cross-linked microscaffolds display more uniform pores with great interconnection than the TPP cross-linked scaffolds and exhibit a water absorption ratio (percent weight change of material due to absorbed water) of around 1,000%, as well as a swelling ratio of about 72%. These spatial structures and physical properties can provide excellent adhesion sites and sufficient nutrients for cell growth. Moreover, both the cancer cell lines (LoVo and MDA-MB-231 cells) cultured on the EDC cross-linked scaffold exhibit good adhesion and spreading. The authors propose that the SF/CS microscaffolds can provide a promising *in vitro* platform for the efficacy prediction and sensitivity screening of various anticancer drugs.

The review article from Prof. Fenelon and colleagues aims to present different applications of pullulan and dextran-based biomaterials for bone tissue engineering (Ahmed Omar et al., 2022). Following the PRISMA guidelines, this article analyzes the published articles from various databases, including Pubmed, Scopus, and Web of Science. This article systematically highlights the strategies for bone regeneration capacity and fabrication processes, the addition of biological elements, and their limitations for bone tissue engineering.

Prof. Chiono and coworkers present the generation of electroconductive hydrogels ECHs based on photo-crosslinked blends of polyethylene glycol diacrylate (PEGDA) and gelatin at different PEGDA:gelatin ratios (1:1, 1.5:1, and 2:1 wt./wt.), and containing poly (3,4-ethylenedioxythiophene):poly (styrene sulfonate) (PEDOT:PSS) (0.0, 0.1, 0.3, and 0.5% w/v) (Testore et al., 2022). The authors demonstrate that using the biocomponent gelatin as a photoinitiator enables its successful incorporation into the hydrogel network. Moreover, adding PEDOTs with reduced photo-crosslinking time increases their surface features and electronic properties, enabling their potential as electrocondutable photo-curable PEGDA-gelatin/PEDOT:PSS hydrogels for cardiac tissue engineering.

Prof. Chen and colleagues demonstrate the approach of generating novel liver organoids *via* micropatterning technique, enabling the reproducible and high throughput formation of fetal liver organoids (Xu et al., 2022). These architectures with uniform morphology and deterministic size recapitulate several critical features, including fetal liver-specific gene and protein expression, glycogen storage, lipid accumulation, and protein secretion.

Prof. Tai and coworkers present the synthesis of a new chiral cross-linker for controlling the matrix morphology of peptide conformational imprints (PCIs) on magnetic particles (PCIMPs) to stabilize their recognition capability (Kanubaddi et al., 2022). These PCIMPs with helical cavities complement the PAP structure to adsorb specifically at the targeted position of papain, showing the best binding parameters to the PAP with Kd = 0.087 μM and Bmax = 4.56 μM.

Prof. Jiang and colleagues demonstrate the encapsulation of bone marrow mesenchymal stem cells (BMSCs) in alginate-chitosan-alginate microcapsules (Yuan et al., 2022). The authors systematically characterize various parameters, suggesting that the designed scaffold displays high porosity and injectability with good collapsibility and compressive strength. Owing to better new bone formation, these microcapsules combined with calcium phosphate bone cement show excellent application prospects as bone engineering materials.

The review article from Prof. X. Wang and colleagues presents the application of SF-based scaffolds for bone regeneration due to excellent biocompatibility, mechanical properties, controllable biodegradability, and structural tunability (Wu et al., 2022). The authors summarize the generation and modification of SF scaffolds and their interactions with tissues and cells toward osteogenesis.

Prof. Tunesi and coworkers propose a highly controllable method to optimize the printability of cross-linked pectin gels with CaCO_3_ (Merli et al., 2022). Specifically, the authors achieve control over the pH as a parameter to generate multiple (pH-dependent) crosslinking kinetics without varying hydrogel composition. Further, the 3D-bioprinted pectin scaffolds show successful results for neural cell culture.

Prof. H. Wang and colleagues exploit the autologous crystallization ability of poly (ε-caprolactone) (PCL) scaffolds to generate nanoneedle arrays (Ren et al., 2022). The surface of nanoneedles coated with polydopamine shows excellent adhesion, spreading, and proliferation abilities of bone marrow mesenchymal stem cells (BMMSCs). The authors demonstrate the positive correlation between the strength of cell-cell interactions, further revealing their scope for designing tissue engineering scaffolds.

Prof. Della Porta and her group demonstrate the fabrication of fibrin scaffolds to populate two different kinds of cells, i.e., BMMSCs and skeletal myoblasts (Scala et al., 2022). Further, the addition of Peripheral blood mononuclear cells (PBMCs) fraction from blood filtration improves the influence of myogenesis. The presence of PBMCs enables the significant downregulation of pro-inflammatory cytokine gene expression. This biomimetic environment provides an excellent tool for investigating the cellular crosstalk and the influence of PBMC in myogenesis.

In the review by Prof. Kankala and colleagues, various kinds of injectable microscale modularized units by biofabrication approaches as ideal delivery vehicles for cells, and various growth factors are described (Duan et al., 2023). The authors emphasize the progress of various microcarriers that potentially pushed the borders of tissue regeneration, highlighting their design, ability to deliver cells, and substantial tissue growth *in situ* and *in vivo* from different viewpoints of materials chemistry and biology. Finally, the perspectives highlighting current challenges and expanding opportunities of these innovative carriers are summarized.

This thematic Research Topic presented several articles exploring the advancements in various innovative synthetic strategies and plausible mechanistic elucidations toward developing polymeric microarchitectures for tissue engineering and drug screening applications. As anticipated, this thematic Research Topic has collected articles presenting various innovative scaffolding systems with considerations of biosafety and degradability along with aspects of current challenges and expanding opportunities of these innovative polymeric carriers for cell delivery applications.
